# Bis(phenanthridinium) hexa­chloridoplatinate(IV) dimethyl sulfoxide disolvate

**DOI:** 10.1107/S1600536809002736

**Published:** 2009-01-28

**Authors:** Selvi Karaca, Mehmet Akkurt, Nasser Safari, Vahid Amani, Orhan Büyükgüngör, Anita Abedi

**Affiliations:** a Department of Physics, Faculty of Arts and Sciences, Erciyes University, 38039 Kayseri, Turkey; b Chemistry Department, Shahid Beheshti University, GC, Tehran, Iran; c Department of Physics, Faculty of Arts and Sciences, Ondokuz Mayıs University, 55139 Samsun, Turkey; d Department of Chemistry, North Tehran Branch, Islamic Azad University, Tehran, Iran

## Abstract

The asymmetric unit of the title compound, (C_13_H_10_N)_2_[PtCl_6_]·2C_2_H_6_OS, contains one independent protonated phenanthridinium cation, half of a centrosymmetric [PtCl_6_]^2−^anion and one dimethyl sulfoxide solvent mol­ecule. Intra­molecular N—H⋯O and inter­molecular C—H⋯Cl hydrogen-bonding inter­actions seem to be effective in the stabilization of the structure.

## Related literature

For related literature, see: Abedi *et al.* (2008 ▶); Amani *et al.* (2008 ▶); Hasan *et al.* (2001 ▶); Hu *et al.* (2003 ▶); Juan *et al.* (1998 ▶); Kalateh *et al.* (2008 ▶); Li & Liu (2003 ▶); Terzis & Mentzafos (1983 ▶); Yousefi, Ahmadi *et al.* (2007 ▶); Yousefi, Teimouri *et al.* (2007*a* ▶,*b* ▶); Zafar *et al.* (2000 ▶); Zordan & Brammer (2004 ▶); Zordan *et al.* (2005 ▶).
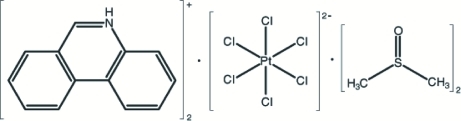


## Experimental

### 

#### Crystal data

(C_13_H_10_N)_2_[PtCl_6_]·2C_2_H_6_OS
*M* *_r_* = 924.50
Orthorhombic, 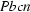

*a* = 24.3695 (11) Å
*b* = 7.9061 (3) Å
*c* = 17.4322 (6) Å
*V* = 3358.6 (2) Å^3^
*Z* = 4
Mo *K*α radiation
μ = 4.81 mm^−1^
*T* = 295 (2) K
0.80 × 0.35 × 0.09 mm

#### Data collection

Stoe IPDS-II diffractometer
Absorption correction: integration (*X-RED32*; Stoe & Cie, 2002 ▶) *T*_min_ = 0.114, *T*_max_ = 0.671
33029 measured reflections
3533 independent reflections
2992 reflections with *I* > 2σ(*I*)
*R*_int_ = 0.106

#### Refinement

*R*[*F* ^2^ > 2σ(*F* ^2^)] = 0.045
*wR*(*F* ^2^) = 0.096
*S* = 1.12
3533 reflections
199 parameters
H atoms treated by a mixture of independent and constrained refinement
Δρ_max_ = 2.20 e Å^−3^
Δρ_min_ = −1.01 e Å^−3^

### 

Data collection: *X-AREA* (Stoe & Cie, 2002 ▶); cell refinement: *X-AREA*; data reduction: *X-RED32* (Stoe & Cie, 2002 ▶); program(s) used to solve structure: *SIR97* (Altomare *et al.*, 1999 ▶); program(s) used to refine structure: *SHELXL97* (Sheldrick, 2008 ▶); molecular graphics: *ORTEP-3 for Windows* (Farrugia, 1997 ▶); software used to prepare material for publication: *WinGX* (Farrugia, 1999 ▶).

## Supplementary Material

Crystal structure: contains datablocks global, I. DOI: 10.1107/S1600536809002736/hg2472sup1.cif

Structure factors: contains datablocks I. DOI: 10.1107/S1600536809002736/hg2472Isup2.hkl

Additional supplementary materials:  crystallographic information; 3D view; checkCIF report

## Figures and Tables

**Table 1:** Hydrogen-bond geometry (Å, °)

*D*—H⋯*A*	*D*—H	H⋯*A*	*D*⋯*A*	*D*—H⋯*A*
N1—HN1⋯O1	0.84 (9)	1.79 (9)	2.621 (8)	169 (8)
C7—H7⋯Cl2^i^	0.93	2.68	3.540 (7)	154

Symmetry code: (i) 
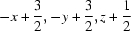
.

**Fig. 1.**
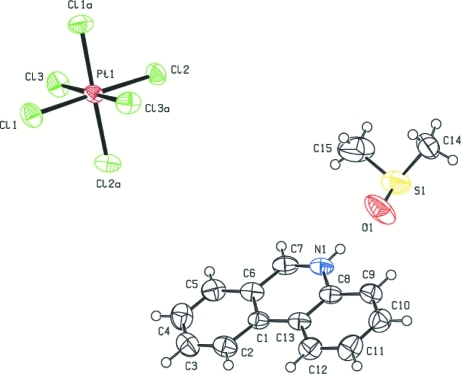
ORTEP-3 view of the title molecule, with the atom-numbering scheme and 50% probability displacement ellipsoids

**Fig. 2.**
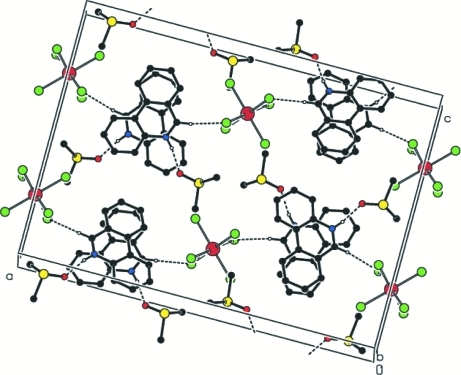
View of the packing and hydrogen bonding interactions. For clarity, H atoms not involved in hydrogen bonds have been omitted.

## supplementary crystallographic information

### Comment

In recent years, there has been considerable interest in proton transfer systems and their structures (Zafar *et al.*, 2000; Abedi *et al.*, 2008). Several proton transfer systems using H_2_[PtCl_6_] with proton acceptor molecules, such as [HpyBr-3]_2_[PtCl_6_].2H_2_O, (II), and [HpyI-3]_2_[PtCl_6_].2H_2_O, (III), (Zordan *et al.*, 2005), [BMIM]_2_[PtCl_6_], (IV), and [EMIM]_2_[PtCl_6_], (V), (Hasan *et al.*, 2001), {(DABCO)H_2_[PtCl_6_]}, (VI), (Juan *et al.*,1998), {*p*-C_6_H_4_(CH_2_ImMe)_2_[PtCl_6_]}, (VII), (Li & Liu, 2003), [het][PtCl_6_].2H_2_O, (VIII), (Hu *et al.*, 2003), [9-MeGuaH]_2_[PtCl_6_].2H_2_O, (IX), (Terzis & Mentzafos, 1983), [HpyCl-3]_3_[PtCl_6_]Cl, (*X*), (Zordan *et al.*, 2004), [2,9-dmphen.H]_2_[PtCl_6_], (XI), (Yousefi *et al.*,  2007), [H_2_DA18C6][PtCl_6_].2H_2_O, (XII), (Yousefi *et al.*, 2007*a*), [2,6-dmpy.H]_2_[PtCl_6_], (XIII), (Amani *et al.*, 2008), [TBA]_3_[PtCl_6_]Cl, (XIV), (Yousefi *et al.*,  2007*b*) and [2,4,6-dmpy.H]_2_[PtCl_6_], (XV), (Kalateh *et al.*, 2008) [where hpy is halo-pyridinium, BMI*M*^+^ is 1-*n*-butyl-3-methylimidazolium, EMI*M*^+^ is1-ethyl-3-methylimidazolium, DABCO is 1,4-diazabicyclooctane, Im is imidazolium, het is 2-(α-hydroxyethyl) thiamine, 9-MeGuaH is 9-methylguaninium, 2,9-dmphen.H is 2,9-dimethyl-1,10-phenanthrolinium, H_2_DA18C6 is 1,10-Diazonia-18-crown-6,2,6-dmpy.H is 2,6-dimethylpyridinium, TBA is tribenzylammonium and 2,4,6-dmpy.H is 2,4,6-dimethylpyridinium] have been synthesized and characterized by single-crystal X-ray diffraction methods. We report herein the synthesis and crystal structure of the title compound, (I).

The asymmetric unit of the title compound (I), (Fig. 1) contains one independent protonated phenanthridinium cation and one half PtCl^2-^_6_ anion, and one dimethyl sulfoxide solvate. The Pt ion has an octahedral coordination (Table 1). In cation, the bond lengths and angles are normal. In PtCl^2-^_6_ anion, the Pt—Cl bond lengths and Cl—Pt—Cl bond angles are also within normal ranges, as in (III) to (XV).

The intramolecular N—H···O and intermolecular C—H···Cl hydrogen bonding interactions (Table 1) seem to be effective in the stabilization of the structure (Fig. 2).

### Experimental

For the preparation of the title compound, (I), a solution of phenanthridine (0.27 g,1.48 mmol) in ethanol (10 ml) was added to a solution of H_2_PtCl_6_.6H_2_O, (0.38 g, 0.74 mmol) in ethanol (10 ml) at room temperature. The suitable crystals for X-ray diffraction experiment were obtained by ethanol diffusion in a solution of orange precipitated in DMSO after one week [yield; 0.51 g, 74.7%, m.p. < 573 K].

### Refinement

The C-bound H-atoms were placed in calculated positions with C—H = 0.93 Å and C—H 0.96 Å, and were included in the refinement in the riding model approximation, with *U*_iso_(H) = 1.2*U*_eq_ (ring C) and *U*_iso_(H) = 1.5*U*_eq_ (methyl C). The N-bound H-atom was found from a difference Fourier map and refined freely. In the final Fourier map, the highest and deepest peaks were located 1.13 and 0.47 Å from atom S1, respectively.

### Crystal data

**Table:**

(C_13_H_10_N)_2_[PtCl_6_]·2C_2_H_6_OS	*F*(000) = 1816
*M**_r_* = 924.50	*D*_x_ = 1.828 Mg m^−^^3^
Orthorhombic, *P**b**c**n*	Mo *K*α radiation, λ = 0.71073 Å
Hall symbol: -P 2n 2ab	Cell parameters from 46774 reflections
*a* = 24.3695 (11) Å	θ = 1.4–27.3°
*b* = 7.9061 (3) Å	µ = 4.81 mm^−^^1^
*c* = 17.4322 (6) Å	*T* = 295 K
*V* = 3358.6 (2) Å^3^	Prism, yellow
*Z* = 4	0.80 × 0.35 × 0.09 mm

### Data collection

**Table:**

Stoe IPDS-2 diffractometer	3533 independent reflections
Radiation source: sealed X-ray tube, 12 x 0.4 mm long-fine focus	2992 reflections with *I* > 2σ(*I*)
plane graphite	*R*_int_ = 0.106
Detector resolution: 6.67 pixels mm^-1^	θ_max_ = 26.8°, θ_min_ = 1.7°
ω scans	*h* = −30→30
Absorption correction: integration (*X-RED32*; Stoe & Cie, 2002)	*k* = −9→10
*T*_min_ = 0.114, *T*_max_ = 0.671	*l* = −22→20
33029 measured reflections	

### Refinement

**Table:**

Refinement on *F*^2^	Primary atom site location: structure-invariant direct methods
Least-squares matrix: full	Secondary atom site location: difference Fourier map
*R*[*F*^2^ > 2σ(*F*^2^)] = 0.045	Hydrogen site location: inferred from neighbouring sites
*wR*(*F*^2^) = 0.096	H atoms treated by a mixture of independent and constrained refinement
*S* = 1.12	*w* = 1/[σ^2^(*F*_o_^2^) + (0.0192*P*)^2^ + 15.6678*P*] where *P* = (*F*_o_^2^ + 2*F*_c_^2^)/3
3533 reflections	(Δ/σ)_max_ = 0.001
199 parameters	Δρ_max_ = 2.20 e Å^−^^3^
0 restraints	Δρ_min_ = −1.01 e Å^−^^3^

### Special details

**Table:**

Geometry. Bond distances, angles *etc*. have been calculated using the rounded fractional coordinates. All su's are estimated from the variances of the (full) variance-covariance matrix. The cell e.s.d.'s are taken into account in the estimation of distances, angles and torsion angles
Refinement. Refinement on *F*^2^ for ALL reflections except those flagged by the user for potential systematic errors. Weighted *R*-factors *wR* and all goodnesses of fit *S* are based on *F*^2^, conventional *R*-factors *R* are based on *F*, with *F* set to zero for negative *F*^2^. The observed criterion of *F*^2^ > σ(*F*^2^) is used only for calculating -*R*-factor-obs *etc*. and is not relevant to the choice of reflections for refinement. *R*-factors based on *F*^2^ are statistically about twice as large as those based on *F*, and *R*-factors based on ALL data will be even larger.

### Fractional atomic coordinates and isotropic or equivalent isotropic displacement parameters (Å^2^)

**Table:**

	*x*	*y*	*z*	*U*_iso_*/*U*_eq_	
N1	0.3026 (2)	0.7872 (7)	0.9147 (3)	0.0450 (17)	
C1	0.2284 (2)	0.9236 (7)	0.8111 (3)	0.0380 (17)	
C2	0.1935 (3)	0.9962 (8)	0.7558 (4)	0.051 (2)	
C3	0.2148 (3)	1.0700 (9)	0.6916 (4)	0.057 (3)	
C4	0.2715 (3)	1.0779 (9)	0.6797 (4)	0.059 (3)	
C5	0.3067 (3)	1.0107 (9)	0.7327 (4)	0.052 (2)	
C6	0.2855 (2)	0.9333 (8)	0.7987 (3)	0.0417 (17)	
C7	0.3209 (3)	0.8621 (8)	0.8539 (4)	0.048 (2)	
C8	0.2463 (3)	0.7702 (8)	0.9301 (3)	0.0403 (17)	
C9	0.2309 (3)	0.6859 (9)	0.9972 (4)	0.049 (2)	
C10	0.1764 (3)	0.6683 (9)	1.0126 (4)	0.056 (2)	
C11	0.1378 (3)	0.7337 (9)	0.9637 (5)	0.060 (3)	
C12	0.1527 (3)	0.8186 (8)	0.8981 (4)	0.051 (2)	
C13	0.2085 (2)	0.8394 (7)	0.8797 (3)	0.0383 (17)	
S1	0.42059 (10)	0.6422 (3)	1.04509 (15)	0.0776 (8)	
O1	0.3643 (2)	0.7031 (10)	1.0318 (4)	0.099 (3)	
C14	0.4292 (4)	0.6686 (12)	1.1452 (5)	0.080 (3)	
C15	0.4641 (5)	0.7984 (15)	1.0151 (6)	0.111 (5)	
Pt1	1.00000	0.67772 (4)	0.25000	0.0358 (1)	
Cl1	0.95124 (7)	0.8825 (2)	0.18305 (10)	0.0533 (5)	
Cl2	1.04739 (6)	0.4687 (2)	0.31590 (9)	0.0509 (5)	
Cl3	1.06735 (7)	0.6805 (2)	0.15568 (9)	0.0525 (5)	
HN1	0.325 (4)	0.753 (11)	0.948 (5)	0.09 (3)*	
H2	0.15570	0.99380	0.76300	0.0610*	
H3	0.19120	1.11580	0.65520	0.0690*	
H4	0.28520	1.12890	0.63560	0.0710*	
H5	0.34440	1.01640	0.72490	0.0620*	
H7	0.35860	0.86920	0.84640	0.0580*	
H9	0.25720	0.64290	1.03050	0.0590*	
H10	0.16530	0.61150	1.05660	0.0670*	
H11	0.10070	0.72030	0.97510	0.0710*	
H12	0.12590	0.86250	0.86580	0.0610*	
H14A	0.40670	0.58810	1.17200	0.1200*	
H14B	0.41840	0.78110	1.15950	0.1200*	
H14C	0.46700	0.65100	1.15850	0.1200*	
H15A	0.46470	0.80150	0.96010	0.1660*	
H15B	0.50040	0.77600	1.03400	0.1660*	
H15C	0.45170	0.90540	1.03450	0.1660*	

### Atomic displacement parameters (Å^2^)

**Table:**

	*U*^11^	*U*^22^	*U*^33^	*U*^12^	*U*^13^	*U*^23^
N1	0.030 (3)	0.053 (3)	0.052 (3)	−0.001 (2)	−0.008 (2)	−0.008 (3)
C1	0.036 (3)	0.037 (3)	0.041 (3)	0.002 (2)	−0.004 (2)	−0.009 (2)
C2	0.043 (3)	0.056 (4)	0.054 (4)	0.007 (3)	−0.005 (3)	−0.007 (3)
C3	0.062 (5)	0.059 (4)	0.051 (4)	0.011 (4)	−0.007 (4)	−0.001 (3)
C4	0.074 (5)	0.057 (4)	0.046 (4)	0.004 (4)	0.013 (4)	0.000 (3)
C5	0.051 (4)	0.061 (4)	0.044 (4)	−0.005 (3)	0.008 (3)	−0.009 (3)
C6	0.034 (3)	0.047 (3)	0.044 (3)	0.005 (3)	0.001 (3)	−0.013 (3)
C7	0.026 (3)	0.057 (4)	0.061 (4)	0.002 (3)	−0.002 (3)	−0.012 (3)
C8	0.036 (3)	0.043 (3)	0.042 (3)	0.002 (3)	−0.001 (3)	−0.013 (3)
C9	0.053 (4)	0.053 (4)	0.042 (3)	−0.002 (3)	−0.006 (3)	−0.004 (3)
C10	0.059 (4)	0.055 (4)	0.053 (4)	−0.005 (4)	0.006 (4)	0.000 (3)
C11	0.040 (4)	0.061 (4)	0.078 (5)	0.000 (3)	0.018 (4)	0.003 (4)
C12	0.035 (3)	0.054 (4)	0.064 (4)	0.005 (3)	0.002 (3)	0.000 (3)
C13	0.032 (3)	0.041 (3)	0.042 (3)	0.004 (2)	−0.001 (2)	−0.013 (3)
S1	0.0602 (13)	0.0891 (16)	0.0836 (15)	−0.0018 (11)	−0.0189 (11)	−0.0056 (12)
O1	0.049 (3)	0.170 (7)	0.077 (4)	0.013 (4)	−0.021 (3)	0.027 (4)
C14	0.072 (6)	0.106 (7)	0.062 (5)	−0.016 (5)	−0.017 (4)	0.030 (5)
C15	0.095 (8)	0.166 (11)	0.071 (6)	−0.051 (8)	0.009 (6)	−0.023 (7)
Pt1	0.0247 (2)	0.0484 (2)	0.0342 (2)	0.0000	0.0005 (1)	0.0000
Cl1	0.0434 (9)	0.0616 (9)	0.0548 (9)	0.0150 (8)	−0.0025 (7)	0.0076 (8)
Cl2	0.0390 (8)	0.0611 (9)	0.0527 (9)	0.0007 (7)	−0.0073 (7)	0.0160 (8)
Cl3	0.0396 (8)	0.0715 (10)	0.0463 (8)	0.0082 (8)	0.0145 (7)	0.0067 (8)

### Geometric parameters (Å, °)

**Table:**

Pt1—Cl1^i^	2.3228 (17)	C8—C13	1.386 (8)
Pt1—Cl2^i^	2.3204 (16)	C9—C10	1.362 (10)
Pt1—Cl3^i^	2.3233 (16)	C10—C11	1.371 (11)
Pt1—Cl3	2.3233 (16)	C11—C12	1.375 (11)
Pt1—Cl1	2.3228 (17)	C12—C13	1.407 (9)
Pt1—Cl2	2.3204 (16)	C2—H2	0.9300
S1—C14	1.770 (9)	C3—H3	0.9300
S1—C15	1.710 (12)	C4—H4	0.9300
S1—O1	1.472 (6)	C5—H5	0.9300
N1—C7	1.293 (9)	C7—H7	0.9300
N1—C8	1.405 (9)	C9—H9	0.9300
N1—HN1	0.84 (9)	C10—H10	0.9300
C1—C6	1.410 (7)	C11—H11	0.9300
C1—C2	1.408 (9)	C12—H12	0.9300
C1—C13	1.452 (7)	C14—H14B	0.9600
C2—C3	1.365 (10)	C14—H14A	0.9600
C3—C4	1.399 (10)	C14—H14C	0.9600
C4—C5	1.368 (10)	C15—H15C	0.9600
C5—C6	1.402 (9)	C15—H15A	0.9600
C6—C7	1.410 (9)	C15—H15B	0.9600
C8—C9	1.398 (9)		
			
Cl1···Cl1^i^	3.331 (2)	C8···O1	3.420 (9)
Cl1···Cl2^i^	3.272 (2)	C8···C6^ix^	3.598 (8)
Cl1···Cl3^i^	3.265 (2)	C8···C9^viii^	3.533 (9)
Cl1···Cl3	3.284 (2)	C8···C1^ix^	3.492 (8)
Cl2···Cl1^i^	3.272 (2)	C9···O1	3.309 (9)
Cl2···Cl3^i^	3.297 (2)	C9···C8^ix^	3.533 (9)
Cl2···Cl3	3.293 (2)	C10···Cl3^xi^	3.646 (7)
Cl2···C7^ii^	3.540 (7)	C13···C6^ix^	3.511 (8)
Cl2···Cl2^i^	3.258 (2)	C2···H12	2.7400
Cl3···Cl2	3.293 (2)	C2···H14A^xii^	2.9200
Cl3···Cl1	3.284 (2)	C4···H10^xii^	3.0400
Cl3···Cl1^i^	3.265 (2)	C10···H3^xiii^	3.0400
Cl3···C10^iii^	3.646 (7)	C12···H2	2.7300
Cl3···Cl2^i^	3.297 (2)	HN1···H9	2.3600
Cl1···H15A^iv^	2.9100	HN1···S1	3.01 (9)
Cl1···H14C^v^	2.9400	HN1···O1	1.79 (9)
Cl1···H2^vi^	2.9400	H2···H12	2.1900
Cl1···H7^iv^	3.0500	H2···H14A^xii^	2.2900
Cl1···H12^vi^	2.8900	H2···C12	2.7300
Cl2···H7^ii^	2.6800	H2···Cl1^vi^	2.9400
Cl2···H15A^ii^	3.1200	H3···C10^xiv^	3.0400
Cl2···H5^ii^	3.0800	H5···H7	2.4400
Cl3···H15C^vii^	3.0700	H5···Cl2^x^	3.0800
Cl3···H10^iii^	3.0000	H5···Cl3^x^	2.9200
Cl3···H5^ii^	2.9200	H7···Cl1^xv^	3.0500
S1···HN1	3.01 (9)	H7···H5	2.4400
O1···N1	2.621 (8)	H7···Cl2^x^	2.6800
O1···C9	3.309 (9)	H9···O1	2.6500
O1···C8	3.420 (9)	H9···HN1	2.3600
O1···HN1	1.79 (9)	H10···C4^xvi^	3.0400
O1···H9	2.6500	H10···Cl3^xi^	3.0000
N1···O1	2.621 (8)	H11···H15B^xvii^	2.4500
C1···C8^viii^	3.492 (8)	H12···C2	2.7400
C1···C4^ix^	3.566 (9)	H12···H2	2.1900
C2···C7^viii^	3.379 (9)	H12···Cl1^vi^	2.8900
C2···C6^viii^	3.573 (9)	H14A···C2^xvi^	2.9200
C3···C6^viii^	3.426 (9)	H14A···H2^xvi^	2.2900
C3···C5^viii^	3.596 (10)	H14B···H15C	2.5200
C4···C1^viii^	3.566 (9)	H14C···H15B	2.5200
C5···C3^ix^	3.596 (10)	H14C···Cl1^xviii^	2.9400
C6···C3^ix^	3.426 (9)	H15A···Cl2^x^	3.1200
C6···C13^viii^	3.511 (8)	H15A···Cl1^xv^	2.9100
C6···C2^ix^	3.573 (9)	H15B···H14C	2.5200
C6···C8^viii^	3.598 (8)	H15B···H11^xix^	2.4500
C7···C2^ix^	3.379 (9)	H15C···H14B	2.5200
C7···Cl2^x^	3.540 (7)	H15C···Cl3^xx^	3.0700
			
Cl1^i^—Pt1—Cl2	89.60 (6)	C9—C10—C11	120.5 (7)
Cl2—Pt1—Cl2^i^	89.18 (6)	C10—C11—C12	121.4 (7)
Cl2—Pt1—Cl3^i^	90.46 (5)	C11—C12—C13	120.1 (6)
Cl1^i^—Pt1—Cl3	89.29 (6)	C1—C13—C8	118.8 (5)
Cl2^i^—Pt1—Cl3	90.46 (5)	C1—C13—C12	124.4 (5)
Cl3—Pt1—Cl3^i^	178.92 (6)	C8—C13—C12	116.9 (5)
Cl1^i^—Pt1—Cl2^i^	178.75 (6)	C1—C2—H2	120.00
Cl1^i^—Pt1—Cl3^i^	89.96 (6)	C3—C2—H2	120.00
Cl2^i^—Pt1—Cl3^i^	90.32 (5)	C2—C3—H3	119.00
Cl2—Pt1—Cl3	90.32 (5)	C4—C3—H3	120.00
Cl1—Pt1—Cl1^i^	91.62 (6)	C3—C4—H4	120.00
Cl1—Pt1—Cl2	178.75 (6)	C5—C4—H4	120.00
Cl1—Pt1—Cl3	89.96 (6)	C6—C5—H5	120.00
Cl1—Pt1—Cl2^i^	89.60 (6)	C4—C5—H5	120.00
Cl1—Pt1—Cl3^i^	89.29 (6)	N1—C7—H7	119.00
O1—S1—C14	103.1 (4)	C6—C7—H7	119.00
O1—S1—C15	107.1 (5)	C8—C9—H9	121.00
C14—S1—C15	98.2 (5)	C10—C9—H9	121.00
C7—N1—C8	122.5 (5)	C11—C10—H10	120.00
C8—N1—HN1	118 (6)	C9—C10—H10	120.00
C7—N1—HN1	119 (6)	C12—C11—H11	119.00
C2—C1—C13	123.3 (5)	C10—C11—H11	119.00
C2—C1—C6	118.0 (5)	C13—C12—H12	120.00
C6—C1—C13	118.7 (5)	C11—C12—H12	120.00
C1—C2—C3	120.4 (6)	S1—C14—H14A	109.00
C2—C3—C4	121.1 (7)	S1—C14—H14B	109.00
C3—C4—C5	120.1 (7)	S1—C14—H14C	110.00
C4—C5—C6	119.5 (6)	H14A—C14—H14B	109.00
C1—C6—C5	120.9 (5)	H14A—C14—H14C	110.00
C5—C6—C7	120.6 (5)	H14B—C14—H14C	110.00
C1—C6—C7	118.5 (5)	S1—C15—H15A	109.00
N1—C7—C6	122.1 (6)	S1—C15—H15B	109.00
C9—C8—C13	122.7 (6)	S1—C15—H15C	109.00
N1—C8—C9	117.9 (6)	H15A—C15—H15B	109.00
N1—C8—C13	119.4 (5)	H15A—C15—H15C	110.00
C8—C9—C10	118.4 (7)	H15B—C15—H15C	109.00
			
C7—N1—C8—C9	179.5 (6)	C4—C5—C6—C1	0.1 (10)
C7—N1—C8—C13	−1.7 (9)	C4—C5—C6—C7	179.6 (6)
C8—N1—C7—C6	0.4 (10)	C1—C6—C7—N1	0.7 (9)
C6—C1—C2—C3	1.4 (9)	C5—C6—C7—N1	−178.8 (6)
C13—C1—C2—C3	−178.4 (6)	N1—C8—C9—C10	−179.8 (6)
C13—C1—C6—C7	−0.6 (8)	C13—C8—C9—C10	1.4 (10)
C2—C1—C13—C8	179.2 (6)	N1—C8—C13—C1	1.7 (8)
C2—C1—C13—C12	1.0 (9)	N1—C8—C13—C12	−179.9 (5)
C6—C1—C13—C8	−0.6 (8)	C9—C8—C13—C1	−179.5 (6)
C6—C1—C13—C12	−178.8 (6)	C9—C8—C13—C12	−1.2 (9)
C2—C1—C6—C5	−0.9 (9)	C8—C9—C10—C11	−0.8 (11)
C2—C1—C6—C7	179.6 (6)	C9—C10—C11—C12	−0.1 (11)
C13—C1—C6—C5	178.9 (6)	C10—C11—C12—C13	0.3 (11)
C1—C2—C3—C4	−1.1 (10)	C11—C12—C13—C1	178.6 (6)
C2—C3—C4—C5	0.2 (11)	C11—C12—C13—C8	0.4 (9)
C3—C4—C5—C6	0.3 (10)		

Symmetry codes: (i) −*x*+2, *y*, −*z*+1/2; (ii) −*x*+3/2, −*y*+3/2, *z*−1/2; (iii) *x*+1, *y*, *z*−1; (iv) *x*+1/2, −*y*+3/2, −*z*+1; (v) −*x*+3/2, *y*+1/2, *z*−1; (vi) −*x*+1, −*y*+2, −*z*+1; (vii) −*x*+3/2, *y*−1/2, *z*−1; (viii) −*x*+1/2, *y*+1/2, *z*; (ix) −*x*+1/2, *y*−1/2, *z*; (x) −*x*+3/2, −*y*+3/2, *z*+1/2; (xi) *x*−1, *y*, *z*+1; (xii) −*x*+1/2, −*y*+3/2, *z*−1/2; (xiii) *x*, −*y*+2, *z*+1/2; (xiv) *x*, −*y*+2, *z*−1/2; (xv) *x*−1/2, −*y*+3/2, −*z*+1; (xvi) −*x*+1/2, −*y*+3/2, *z*+1/2; (xvii) *x*−1/2, −*y*+3/2, −*z*+2; (xviii) −*x*+3/2, *y*−1/2, *z*+1; (xix) *x*+1/2, −*y*+3/2, −*z*+2; (xx) −*x*+3/2, *y*+1/2, *z*+1.

### Hydrogen-bond geometry (Å, °)

**Table:**

*D*—H···*A*	*D*—H	H···*A*	*D*···*A*	*D*—H···*A*
N1—HN1···O1	0.84 (9)	1.79 (9)	2.621 (8)	169 (8)
C7—H7···Cl2^x^	0.93	2.68	3.540 (7)	154

Symmetry codes: (x) −*x*+3/2, −*y*+3/2, *z*+1/2.
